# Improving prodigiosin production by transcription factor engineering and promoter engineering in *Serratia marcescens*

**DOI:** 10.3389/fmicb.2022.977337

**Published:** 2022-08-03

**Authors:** Xuewei Pan, Jiajia You, Mi Tang, Xian Zhang, Meijuan Xu, Taowei Yang, Zhiming Rao

**Affiliations:** Key Laboratory of Industrial Biotechnology of the Ministry of Education, Laboratory of Applied Microorganisms and Metabolic Engineering, School of Biotechnology, Jiangnan University, Wuxi, China

**Keywords:** *Serratia marcescens*, prodigiosin, DNA-binding response regulator OmpR, transcription factor engineering, promoter engineering

## Abstract

Prodigiosin (PG), a red linear tripyrrole pigment produced by *Serratia marcescens*, has attracted attention due to its immunosuppressive, antimicrobial, and anticancer properties. Although many studies have been used to dissect the biosynthetic pathways and regulatory network of prodigiosin production in *S. marcescens*, few studies have been focused on improving prodigiosin production through metabolic engineering in this strain. In this study, transcription factor engineering and promoter engineering was used to promote the production of prodigiosin in *S. marcescens* JNB5-1. Firstly, through construing of a Tn5G transposon insertion library of strain JNB5-1, it was found that the DNA-binding response regulator BVG89_19895 (OmpR) can promote prodigiosin synthesis in this strain. Then, using RNA-Seq analysis, reporter green fluorescent protein analysis and RT-qPCR analysis, the promoter P17 (P*_RplJ_*) was found to be a strong constitutive promoter in strain JNB5-1. Finally, the promoter P17 was used for overexpressing of prodigiosin synthesis activator OmpR and PsrA in strain JNB5-1 and a recombinant strain PG-6 was obtained. Shake flask analysis showed that the prodigiosin titer of this strain was increased to 10.25 g/L, which was 1.62-times that of the original strain JNB5-1 (6.33 g/L). Taken together, this is the first well-characterized constitutive promoter library from *S. marcescens*, and the transcription factor engineering and promoter engineering can be also useful strategies to improve the production of other high value-added products in *S. marcescens*.

## Introduction

Prodigiosin (PG), a red linear tripyrrole pigment, is the most prominent member of the prodiginine family and is mainly produced by *Serratia marcescens*. Due to its important activities in antimalarial, antibacterial, antifungal, antiprotozoal and immunosuppressant, prodigiosin has received widespread attention in the last few decades (Williamson et al., 2006a). And compared with the traditional chemical methods, the production of prodigiosin by microbial fermentation is more economical and environmentally friendly and hence has recently attracted lots of interest. However, although many studies have been used to dissect the biosynthetic pathways and regulatory network of prodigiosin production in *S. marcescens* (Horng et al., 2002, 2010; Coulthurst et al., 2006; Williamson et al., 2006b; Shanks et al., 2013, 2017; Stella and Shanks, 2014; Lee et al., 2017; Stella et al., 2018; Brothers et al., 2019; Pan et al., 2020, 2021, 2022), few studies have been used to improve the efficiency of prodigiosin synthesis through metabolic engineering in this strain.

In the past, in order to improve the ability of native *S. marcescens* strains to synthesize of prodigiosin, lots of studies were focus on the optimization of fermentation parameters such as medium composition and pH (Chang et al., 2011; Fender et al., 2012), temperature (Elkenawy et al., 2017) and incubation period (Elkenawy et al., 2017), but it is still a challenge for high-efficiency production of prodigiosin for commercial purposes. Hence, in addition to the optimization of fermentation process, other methods are also needed to identify the strains with high-yield prodigiosin-producing. Promoter engineering is a method widely used to enhance gene expression at the transcription level. And to improve the yield of target products, numbers of homologous or heterologous promoters have been developed for constitutive or inducible gene expression in model strain such as *Escherichia coli* (Song et al., 2015; Tang et al., 2020), *Bacillus subtilis* (Cui et al., 2019; Fu et al., 2022), and *Corynebacterium glutamicum* (Huang et al., 2021; Liu et al., 2022) and non-model strain such as *Streptococcus thermophilus* (Kong et al., 2019), *Corynebacterium ammoniagenes* (Hou et al., 2019), and *Schlegelella brevitalea* (Ouyang et al., 2020). However, the choice of strong constitutive natural promoters in prodigiosin-producing strain *S. marcescens* is still limited. Due to its efficient application in altering gene transcription to obtain beneficial cellular phenotype, transcription factor engineering has also been used to improve the yield of the target product in recent years (Zhang et al., 2015; Li et al., 2018; Deng et al., 2021). And in our previous studies, we have identified that the *pig* gene cluster essential for prodigiosin synthesis is positively regulated by transcription factor PsrA in *S. marcescens* (Pan et al., 2022). Therefore, it probably can be improving the prodigiosin production in *S. marcescens* strains through transcription factor engineering and promoter engineering.

*Serratia marcescens* JNB5-1 was a strain isolated from soil samples, and it produced a relatively large amount of prodigiosin (Pan et al., 2021). In this study, the prodigiosin production of *S. marcescens* JNB5-1 was further improved by promoter engineering and transcription factor engineering. Firstly, by constructing a Tn5G transposon insertion mutant library, the DNA-binding response regulator OmpR was identified to positively regulates prodigiosin synthesis in strain JNB5-1. Secondly, based on the systematic analysis of time-series transcriptome data of the strain JNB5-1 in different conditions, a strong constitutive promoter was identified. Finally, through improving the expression level of the transcription regulator OmpR and PsrA using the identified P17 promoter, the synthesis of prodigiosin was significantly enhanced in the recombinant strain PG-6. Here the strong constitutive promoter identified and the metabolic engineering strategy used should be valuable for the optimization of pathways for the biosynthesis of other high value-added products in *S. marcescens*.

## Materials and methods

### Bacterial strains, plasmids, and growth conditions

*Serratia marcescens* JNB5-1 is a prodigiosin producing strain isolated from soil samples (Pan et al., 2021). Mutant SK6-35, a prodigiosin production mutant, was isolated from a Tn5G transposon insertion mutant library of strain JNB5-1. PG-1, PG-2, PG-3, PG-4, PG-5 and PG-6 are prodigiosin producing recombinant strains constructed by overexpression of *ompR* and/or *psrA* genes under the control of its own promoter or P17 promoter in strain JNB5-1. *E. coli* DH5α and S17-1λpair were used for plasmid construction. The *E. coli* strains were grown in LB medium at 37°C, and the *S. marcescens* strains were grown in LB medium (yeast extract 0.5%, tryptone 1%, and NaCl 1%) or fermentation medium (sucrose 2%, beef extract 1.5%, CaCl_2_ 1%, L-proline 0.75%, MgSO_4_·7H_2_O 0.02%, and FeSO_4_·7H_2_O 0.006%) at 30°C. Whenever necessary, the medium was added at defined concentrations as follows for strains cultivation. For the cultivation of *E. coli* strains, ampicillin at 50 μg/ml, apramycin at 50 μg/ml or gentamicin at 10 μg/ml were used. For the cultivation of *S. marcescens* strains, ampicillin at 150 μg/ml, apramycin at 50 μg/ml, or gentamicin at 50 μg/ml were used. Bacterial strains and plasmids used in this study are listed in Table 1.

**Table 1:** Strains and plasmids used in this study.

Strain or plasmid	Description	Source
***E. coli* strains**		
DH5α	*hsdR recA lacZYAF80 lacZ*ΔM15	BRL
S17-1	F− *recA hsdR* RP4-2 (Tc::Mu) (Km::Tn7) lysogenized with λpir phage	Laboratory collection
***Serratia marcescens* strains**		
JNB5-1	*S. marcescens* wild type strain	Pan et al. (2020)
SK6-35	*ompR*::Gm^R^ mutant of JNB5-1, prodigiosin producing mutant	This study
SK6-35/pXW2010	Mutant SK6-35 containing plasmid pXW2010	This study
ΔOmpR	*ompR* deleted mutant of *S. marcescens* JNB5-1	This study
ΔOmpR/pXW2010	Mutant ΔOmpR containing plasmid pXW2010	This study
PG-1	Prodigiosin producing recombinant strain constructed by overexpression of *psrA* gene under the control of its own promoter P*_PsrA_* in strain JNB5-1	This study
PG-2	Prodigiosin producing recombinant strain constructed by overexpression of *ompR* gene under the control of its own promoter P*_OmpR_* in strain JNB5-1	This study
PG-3	Prodigiosin producing recombinant strain constructed by overexpression of *ompR* and *psrA* genes under the control of their own promoters in strain JNB5-1	This study
PG-4	Prodigiosin producing recombinant strain constructed by overexpression of *psrA* gene under the control of the promoter P17 (P*_RplJ_*) in strain JNB5-1	This study
PG-5	Prodigiosin producing recombinant strain constructed by overexpression of *ompR* gene under the control of the promoter P17 (P*_RplJ_*) in strain JNB5-1	This study
PG-6	Prodigiosin producing recombinant strain constructed by overexpression both of *ompR* and *psrA* genes under the control of the promoter P17 (P*_RplJ_*) in strain JNB5-1	This study
**Plasmids**		
pRK2013Tn5G	Tn5G carrying plasmid, Km^R^Gm^R^	Nunn and Lory (1992)
pMD18T	Cloning vector, 2,692 bp, Ap^R^, *lacZ*	TaKaRa
pXW2010	*ompR* gene driven by P_lac_ promoter cloned in pUCP18, Ap^R^	This study
pUCP18	Broad-host-range shuttle vector, Ap^R^	Schweizer (1991)
pUTKm	Tn5-based delivery plasmid with Km^R^Amp^R^	Herrero et al. (1990)

### Identification of the Tn5G insertion site in mutant SK6-35

**Figure 1:**
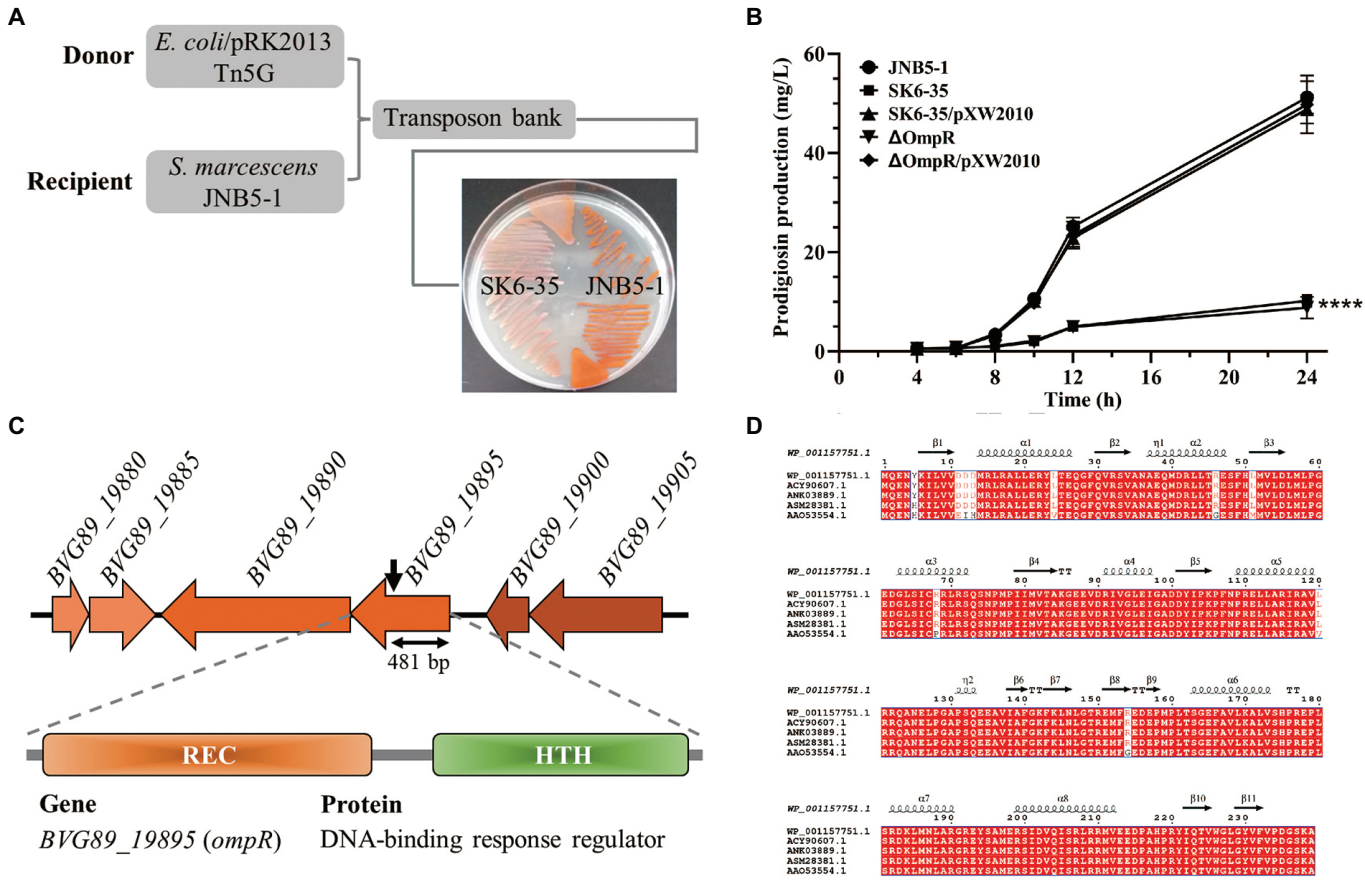
OmpR is a prodigiosin synthesis activator in strain JNB5-1. **(A)** A prodigiosin producing mutant SK6-35 was identified by Tn5G transposon insertion mutation. **(B)** Prodigiosin production analysis of strains JNB5-1, SK6-35, SK6-35/pXW2010, ΔPsrA, and ΔPsrA/pXW2010. JNB5-1 is a wild-type *Serratia marcescens*, SK6-35 is an *ompR* disrupted mutant, ΔOmpR is an *ompR* deleted mutant, SK6-35/pXW2010 and ΔOmpR/pXW2010 are *ompR* complemented strains. The experiment was performed independently three times. Error bars indicate standard deviations. One-way analysis of variance (ANOVA) was used to examine the mean differences between the data groups. ****p < 0.001. **(C)** Genetic loci identified in mutant SK6-35. (Upper) Genetic map of the disrupted gene *BVG89_19895* (*ompR*) and its surrounding genes. The Tn5G insertion site is indicated by black arrow points. (Middle) The domain organization of the BVG90_19895 protein. (Lower) BVG90_19895 is a DNA-binding response regulator. **(D)** Multiple sequence alignment of OmpR homologies. The sequences used for analysis were OmpR homologies from *S. marcescens* (ASM28381.1), *E. coli* (ANK03889.1), *Klebsiella pneumoniae* (WP_001157751.1), *Salmonella enterica* subsp. *enterica serovar Typhimurium* (ACY90607.1), and *Yersinia enterocolitica* (AAO53554.1).

Tn5G transposon was used to mutate *S. marcescens* JNB5-1 to identify prodigiosin-producing mutants and prodigiosin synthesis regulator in strain JNB5-1 as described previously (Nunn and Lory, 1992; Pan et al., 2020). In brief, as shown in Figure 1A *E. coli*/pRK2013 Tn5G was used as the donor strain and *S. marcescens* JNB5-1 was used as the recipient strain. After mating, the mutant bank was plated onto LB agar medium with 50 μg/ml gentamicin and 50 μg/ml ampicillin, and a mutant strain SK6-35 with significantly decreased prodigiosin production was isolated. Then, to identify the Tn5G insertion site in the strain SK6-35, inverse PCR method was performed as described previously (Wang et al., 1996). In brief, as shown in Supplementary Figure S1, the genomic DNA of strain SK6-35 containing Tn5G transposon was extracted, and completely digested by the restriction nuclease *Taq*I. Then the DNA molecules were subjected to self-ligation and amplified using the primers OTn1 and OTn2 as listed in Supplementary Table S1. The PCR product was cloned into the pMD18T vector for sequencing, and the sequence obtained were compared with the NCBI GenBank database to identify the Tn5G transposon insertion site. The domains of the DNA-binding response regulator BVG89_19895 was identified using the online software CD-search. For complementation experiments, the target gene *BVG89_19895* (*ompR*) identified was amplified, cloned into the pUCP18 plasmid to obtain recombinant plasmid pXW2010 and introduced to strains SK6-35 and ΔOmpR.

### Transcriptome analysis

Constitutive promoters should have the characteristics that there has no significant difference in inducing gene expression at different cell growth stages and under different conditions. Hence, to identify strong constitutive promoters in *S. marcescens*, strain JNB5-1 were grown to early logarithmic growth phase at 4 h and mid-logarithmic growth phase at 12 h in LB medium and fermentation medium prior to harvesting. A volume of 1 ml of the collected cells was then frozen in liquid nitrogen, treated with RNAprep pure kit (Tiangen) to extract total bacterial RNA, and delivered to Genewiz (Genewiz, South Plainfield, NJ) for transcriptome resequencing analysis. With Ribo-Zero rRNA Removal Kit (Illumina, San Diego, CA, United States), the total RNA extracted from the bacteria was subjected to rRNA removal to obtain mRNA. The obtained mRNA was used as the template for DNA synthesis. Illumina HiSeq platform was used for cDNA library sequencing. The genome of *S. marcescens* WW4 (NC_020211.1) was used as the reference for annotation. The FPKM values were used to rank the genes within each sample from highest to lowest expression, and the top 4.1% highly expressed genes (200 genes) in each condition were chosen for further expression analysis to identified strong constitutive promoters in *S. marcescens*.

### Construction of plasmids and strains for promoter characterization

To characterize the identified promoters as shown in Table 2, the promoter regions of these 32 strongly expressed genes under both culturing conditions and the promoter of *bla* gene were amplified by PCR. The PCR products were then cloned into the pUCP18 plasmid harboring the *egfp* reporter gene using the DNA assembler method to obtain recombinant plasmids pUCP18-promoter-egfp and electroporated into strain JNB5-1 to construct recombinant strains. Primers used for plasmid construction and promoter characterization are listed in Supplementary Table S1.

**Table 2:** Identified genes with high level expression from RNA-seq data.

Gene	Promoter	Downstream product	Average FPKM
*ahpC*	1	Alkyl hydroperoxide reductase	3612.45
*clpP*	2	ATP-dependent Clp protease	1151.7725
*cpxP*	3	Cpx response inhibitor	1919.7225
*cspC*	4	Stress protein, member of the CspA-family	8650.905
*eno*	5	Enolase	1959.8025
*ftsZ*	6	Cell division protein FtsZ	1703.47
*gltA*	7	Citrate synthase	12997.47
*lpp*	8	Murein lipoprotein	6366.5075
*lpxC*	9	UDP-3-O-acyl N-acetylglucosamine deacetylase	2541.38
*nlpI*	10	Lipoprotein	1242.08
*ompA*	11	Outer membrane protein A	21640.7025
*ompC*	12	Outer membrane porin protein C	4732.635
*ompN*	13	Outer membrane pore protein	10778.6525
*ompW*	14	Outer membrane protein W	5126.42
*ompX*	15	Outer membrane protein X	6679.55
*raiA*	16	Cold shock protein	9965.2425
*rplJ*	17	50S ribosomal subunit protein L10	5684.93
*rpoB*	18	RNA polymerase, beta subunit	1454.715
*rpoH*	19	RNA polymerase, sigma H factor	1774.61
*rpsA*	20	30S ribosomal subunit protein S1	2618.3375
*rpsF*	21	30S ribosomal subunit protein S6	5404.0175
*rpsM*	22	30S ribosomal subunit protein S13	5304.5475
*SMWW4_v1c12140*	23	Hypothetical protein	1975.2125
*SMWW4_v1c29250*	24	Hypothetical protein	2818.635
*sodB*	25	Superoxide dismutase	2468.105
*tpiA*	26	Triosephosphate isomerase	2013.625
*trxA*	27	Thioredoxin 1	1508.045
*uspG*	28	Universal stress protein UP12	1753.1375
*ybaY*	29	Outer membrane lipoprotein	8141.1775
*yccA*	30	HflBKC-binding inner membrane protein	1318.955
*yfiD*	31	Autonomous glycyl radical cofactor	3249.205
*ygdI*	32	Putative lipoprotein	2606.52

### Measurement of fluorescence intensity of eGFP

The fluorescence values of eGFP of different strains were measured to further identify strong constitutive promoters in *S. marcescens* as described previously (You et al., 2022). In brief, strain JNB5-1/pUCP18 and recombinant strains containing plasmids pUCP18-promoter-egfp were inoculated in sterile 96 black-well plates (corning 3603) containing 200 μl LB medium and incubated at 37°C for 10 h. Then eGFP fluorescence was determined at the end of the incubation with a microplate Multi-Mode Reader (BIOTEK, Cytation 3) at the absorption wavelengths of 490 nm and 530 nm.

### Promoter characterization *via* RT-qPCR analysis

RT-qPCR assay was performed to further characterize the identified strong constitutive promoters as described previously (Ouyang et al., 2020; Pan et al., 2020). In brief, to confirm that the promoters of *cspC* (P4), *ompA* (P11), *rplJ* (P17), *rpsA* (P20), *rpsF* (P21), and *rpsM* (P22) genes are strong constitutive promoters in *S. marcescens*, A volume of 1 ml of the cultures of strains JNB5-1 cultured in LB medium carrying the recombinant plasmids pUCP18-promoter-egfp were collected at early logarithmic growth phase at 4 h, mid-logarithmic growth phase at 12 h and stationary growth phase at 24 h. Total RNA of the collected cells was then extracted using the RNAprep pure Cell/Bacteria Kit (Tiangen). After treating with DNase I (Promega) for 30 min at room temperature, 0.5 μg of the total bacterial RNA was subjected to reverse transcription to synthesize cDNA using the HiScript II Q RT SuperMix Kit (Vazyme). To ensure that there was no chromosomal DNA contamination in each RNA sample, a no-reverse transcription control of each sample was carried out and any samples with detected chromosomal DNA contamination were excluded before experimentation. Then, cDNA of each sample was mixed with forward and reverse primers and mixture was subjected to RT-qPCR analysis using the ChamQ Universal SYBR qPCR master mix kit (Vazyme). RNA from three biological replicates were analyzed and three technical replicates were performed. The 16S rRNA protein-encoding gene was used as an internal control for promoter characterization. The expression level of the *egfp* gene under promoters P4, P11, P17, P20, P21, and P22 were normalized by the expression of the internal control. Data were analyzed using 2^–ΔΔCt^ method.

### Construction of strains ΔOmpR, PG-1, PG-2, PG-3, PG-4, PG-5, and PG-6

The *ompR* gene of strain JNB5-1 was deleted using a gene replacement method as described previously (Pan et al., 2020). In brief, after the upstream and downstream DNA fragments of *ompR* gene and the DNA fragment of *aacC3* resistance gene were amplified, the *aacC3* gene was integrated into the middle of the upstream and downstream fragments of the *ompR* gene by overlap extension PCR. The obtained PCR products were cloned into the pUTKm vector to obtain recombinant plasmid. The resulted plasmid was transformed into *E. coli* S17-1, and introduced into the strain JNB5-1 by conjugation to knock out the *ompR* gene. To construct prodigiosin producing strains PG-1, PG-2, PG-3, PG-4, PG-5, and PG-6, the DNA fragments containing *psrA* and/or *ompR* genes under the control of its own promoter or P17 promoter were amplified by PCR. The obtained DNA fragments were then cloned into pUCP18 plasmid to obtain recombinant plasmids, and the resulted plasmids were electroporated into JNB5-1 to construct strain PG-1, PG-2, PG-3, PG-4, PG-5, and PG-6. Primers used for *ompR* gene deletion and PG-1, PG-2, PG-3, PG-4, PG-5 and PG-6 strains construction are listed in Supplementary Table S1.

### Prodigiosin production assays in shake flask fermentation

The ability of strains JNB5-1, SK6-35, SK6-35/pXW2010, ΔOmpR, ΔOmpR/pXW2020, PG-1, PG-2, PG-3, PG-4, PG-5 and PG-6 to produce prodigiosin was determined in shake-flask fermentation in LB medium or fermentation medium by acidified ethanol and absorbance measurement as previously described (Pan et al., 2019). Briefly, after collecting samples at the indicated time intervals, the amount of prodigiosin produced by different strains was calculated according to the standard curve: Y = 1.1936X − 0.001 (Y indicates the wavelength of samples measured at A_535_ after the fermentation broth was dissolved in acid ethanol at pH 3.0; X indicates the amount of prodigiosin produced by strains, for which 1 unit equals 10 mg/L). Due to we have previously confirmed that the strain JNB5-1 can obtain the highest production of prodigiosin in LB medium and fermentation medium at 24 h and 72 h, respectively (Pan et al., 2019, 2020), the final fermentation end time of different strains in LB medium and fermentation medium was 24 h and 72 h, respectively. Experiments were independently replicated three times.

### Statistical analysis

Experiments in this study were independently replicated at least three times, and data are expressed as means and standard deviations (SDs). Student’s *t*-test or one-way ANOVA was used for comparing statistical difference between the groups of experimental data.

## Results

### Identification of a regulator OmpR that positively controls prodigiosin synthesis in *Serratia marcescens* JNB5-1

To identify genes that regulate prodigiosin production and improve the efficiency of prodigiosin synthesis through metabolic engineering in strain JNB5-1, a Tn5G transposon insertion library was constructed using *E. coli*/pRK2013 Tn5G as the donor strain and *S. marcescens* JNB5-1 as the recipient strain. As shown in Figure 1A, a mutant SK6-35 with severely reduced prodigiosin synthesis was isolated (Figure 1A). Furthermore, shake flask fermentation analysis in LB medium showed that the SK6-35 mutant could synthesize 10.21 mg/L of prodigiosin after 24 h of fermentation, which was only 0.20 times that synthesized by wild-type strain JNB5-1 (51.22 mg/L; *p* < 0.001; Figure 1B).

With inverse PCR and sequencing, the target gene inserted by Tn5G transposon in strain SK6-35 was identified as *BVG89_19895* encoding a DNA-binding response regulator of 240 amino acids (Figure 1C). At the amino acid level, the identified gene *BVG89_19895* encoding protein BVG89_19895 shares 100% identify with a predicted two-component system response regulator OmpR of a sequenced *S. marcescens* strain, Db11 (HG326223.1). This protein consists of a response regulator receiver (REC) domain at its N terminus and a helix-turn-helix (HTH) domain at its C terminus (Figure 1C), and is 99.58, 99.58, 99.58, and 96.23% identical to proven OmpR proteins of *E. coli* O25b: H4 (ANK03889.1; Dupont et al., 2017), *Klebsiella pneumoniae* KPNIH1 (WP_001157751.1; Ramage et al., 2017), *Salmonella enterica* subsp. *enterica serovar Typhimurium* str. 14028S (ACY90607.1; Jayeola et al., 2020), and *Yersinia enterocolitica* strain Ye9 (AAO53554.1; Jaworska et al., 2018), respectively (Figure 1D). Since the high similarity to previously studied OmpR proteins from other bacterial genera, we therefore referred to the BVG89_19895 open reading frame as OmpR. In the complementation experiment, the intact *ompR* gene was introduced into the SK6-35 mutant, and a complementary strain, *S. marcescens* SK6-35/pXW2010 had increased prodigiosin synthesis significantly in the mutant SK6-35 (Figure 1B). These results suggested that OmpR was possibly associated with the regulation of prodigiosin synthesis in strain JNB5-1.

To further confirm the function of the *ompR* gene in prodigiosin synthesis in strain JNB5-1, a mutant strain ΔOmpR completely deleted of the *ompR* gene was generated, and its ability to synthesize prodigiosin in LB medium was analyzed. Results showed that compared with wild-type strain JNB5-1, the prodigiosin production of the mutant ΔOmpR was significant decreased and was similar to that of *ompR*-disrupted mutant SK6-35 (Figure 1B). Taken together, these results suggested that OmpR functions as a prodigiosin synthesis activator in strain JNB5-1, and how OmpR regulates prodigiosin synthesis in strain JNB5-1 will be described elsewhere.

### Rational selection of putative strong constitutive promoters in *Serratia marcescens*

**Figure 2:**
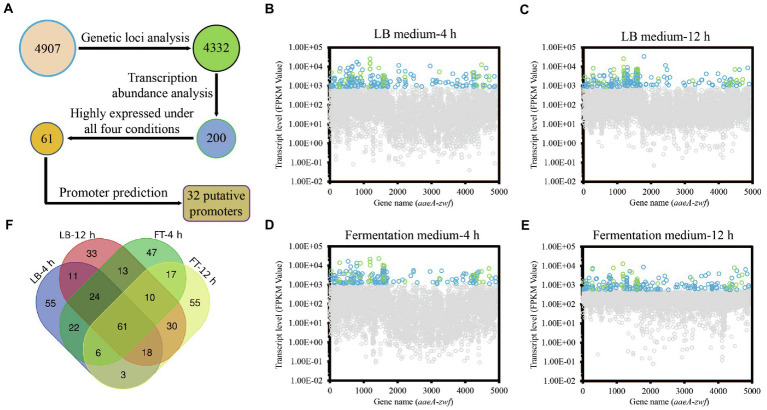
Rational selection of strong constitutive promoters in *Serratia marcescens*. **(A)** The flowchart for selection of strong constitutive promoters from the transcriptomic data. **(B)** Expression levels of the *S. marcescens* genes in LB medium at 4 h. **(C)** Expression levels of the *S. marcescens* genes in LB medium at 12 h. **(D)** Expression levels of the *S. marcescens* genes in fermentation medium at 4 h. **(E)** Expression levels of the *S. marcescens* genes in fermentation medium at 12 h. For **(B–E)**, light blue indicates the top 200 highly expressed genes in each condition, light green indicates the putative strong constitutive promoters encoding gene after rational selection. **(F)** Venn diagram of the number of genes expression highly under all four conditions by RNA-seq. FT-4 h indicates the top 200 highly expressed genes in fermentation medium at 4 h. FT-12 h indicates the top 200 highly expressed genes in fermentation medium at 12 h. LB-4 h indicates the top 200 highly expressed genes in LB medium at 4 h. LB-12 h indicates the top 200 highly expressed genes in LB medium at 12 h.

Promoter engineering was widely used to enhance gene expression at the transcription level and achieved high yields production of target metabolites in different microorganisms (Tang et al., 2020; Fu et al., 2022; Liu et al., 2022). To improve the production of prodigiosin in *S. marcescens*, a panel of strong promoters must be available for fine-tune gene expression in *S. marcescens*. However, the choice of strong constitutive promoters in *S. marcescens* is still limited. Recently, a method based on transcriptome and biochemical experiments has been developed to conveniently screening of strong constitutive promoters in *Schlegelella brevitalea* (Ouyang et al., 2020), *C. ammoniagenes* (Hou et al., 2019), *S. thermophilus* (Kong et al., 2019), and *Streptomyces albus* (Luo et al., 2015). To address the problem that the available strong constitutive promoters are rather limited in *S. marcescens*, the transcriptional profiles of all 4,907 genes in the genome of strain JNB5-1 was conducted by RNA-seq analysis, and the promoters were sorted by their expression levels to identify strong constitutive promoters based on two target culturing mediums (LB medium and fermentation medium) at two defined time points (4 and 12 h; Figures 2A–E). Results show that a total of 4,332 genes were identified from the transcriptomic data (Figure 2A). Only the top 200 highly expressed genes in each condition were chosen, and 61 genes were identified that highly expressed under all four conditions (Figure 2F; Supplementary Table S2). Furthermore, functional analysis was performed to remove genes whose expression profiles were potentially not stable, although these genes were qualified according to our current conditions. One of the groups was the regulators, whose expression profiles are usually condition-dependent and influenced by a highly dynamic complex regulatory network in bacterial (Fineran et al., 2005; Sun et al., 2022). Also, in many cases, genes in the same operon have different expression profiles, which means that there is a potential risk of selecting the promoter of the operon as a constitutive promoter. Hence, another group of the genes removed were the genes that in the operon, and finally a total of 33 genes were kept after removing the genes encoding regulators and the genes in the operon. Among these 33 genes, the promoter region of the *rpsE* gene is very short, hence a total of 32 promoter regions of the genes as shown in Table 2 were cloned into the pUCP18 plasmid harboring an *egfp* gene using recombineering (Table 2). In addition, the promoter P_AmpR_ previously used in strain JNB5-1 was cloned into the pUCP18 plasmid as control (Pan et al., 2021). The promoter regions of these 33 genes are a defined region between the highly expressed gene and its upstream gene. All of the above constructed plasmids were validated by sequencing and then transformed into *S. marcescens* strain JNB5-1.

### Evaluation of the identified putative strong constitutive promoters in *Serratia marcescens*

**Figure 3:**
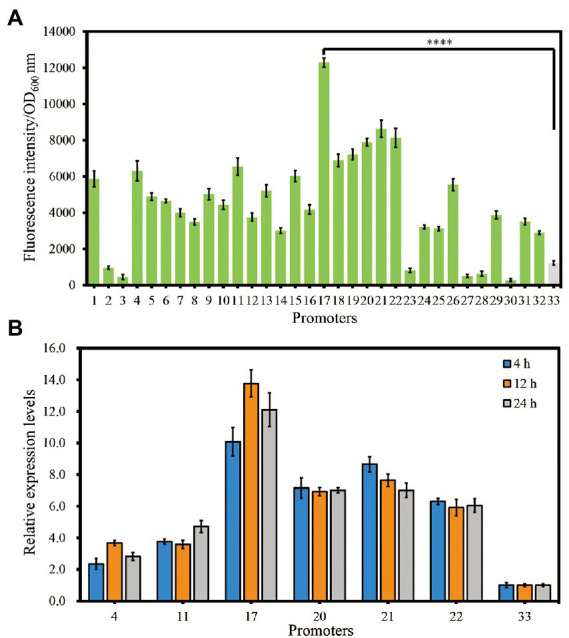
Evaluation of the identified putative strong constitutive promoters in strain JNB5-1 *via* eGFP reporter and RT-qPCR analysis. **(A)** Evaluation of the selected promoters using an eGFP reporter. **(B)** RT-qPCR assays of the selected promoters. The promoters 1, 2, 3, 4, 5, 6, 7, 8, 9, 10, 11, 12, 13, 14, 15, 16, 17, 18, 19, 20, 21, 22, 23, 24, 25, 26, 27, 28, 29, 30, 31, 32, and 33 indicates the promoter of the *ahpC*, *clpP*, *cpxP*, *cspC*, *eno*, *ftsZ*, *gltA*, *lpp*, *lpxC*, *nlpI*, *ompA*, *ompC*, *ompN*, *ompW*, *ompX*, *raiA*, *rplJ*, *rpoB*, *rpoH*, *rpsA*, *rpsF*, *rpsM*, *SMWW4_v1c12140*, *SMWW4_v1c29250*, *sodB*, *tpiA*, *trxA*, *uspG*, *ybaY*, *yccA*, *yfiD*, *ygdI*, and *bla* gene, respectively. For **(A)** and **(B)**, the experiment was performed independently three times. Error bars indicate standard deviations. One-way analysis of variance (ANOVA) was used to examine the mean differences between the data groups. ****p < 0.001.

To examine whether the putative strong constitutive promoters obtained by systematic analyses were reliable, growth-normalized fluorescence intensities of different strains were measured using a microplate assay. As shown in Figure 3A, the results showed that we successfully isolated 32 endogenous promoters with different activities. Among them, 26 promoters (P1, P4, P5, P6, P7, P8, P9, P10, P11, P12, P13, P14, P15, P16, P17, P18, P19, P20, P21, P22, P24, P25, P26, P29, P31, and P32) showed increased activities with 2.38-to 10.14-fold enhancement compared with that of promoter P33 (P_AmpR_; Figure 3A). To the best of our knowledge, this is the first well-characterized strong constitutive promoter library in *S. marcescens*.

Furthermore, RT-qPCR analysis was used to quantified gene expression levels to confirmed the promoter activities in a time-course study. The 16S rRNA protein-encoding gene was used as an internal control as described previously (Wilf et al., 2011). Promoters P4, P11, P17, P20, P21, and P22 with stronger activity than P_AmpR_ and P_AmpR_ itself were used for RT-qPCR experiment. All seven promoters were analyzed under the LB medium at three different time points: 4 h, 12 h and 24 h. Result showed that all six promoters showed higher expression levels than P_AmpR_ at all three time points (Figure 3B). This result further confirmed that the strengths of these promoters. Among them, consistent with the result of fluorescence intensities test, the promoter P17 showed highest strength in RT-qPCR experiment (Figures 3A,B). Hence, in our next study, we will try to overexpress the key gene of prodigiosin synthesis with promoter P17 to improve the ability of strain JNB5-1 to synthesize prodigiosin.

### Improving prodigiosin production by transcription factor engineering and promoter engineering in strain JNB5-1

**Figure 4:**
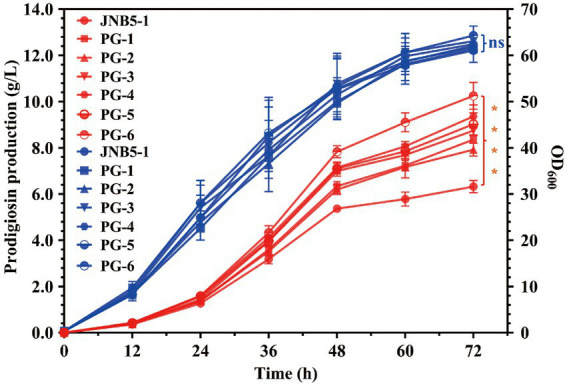
Prodigiosin production and cell growth curve of strains JNB5-1, PG-1, PG-2, PG-3, PG-4, PG-5, and PG-6 in fermentation medium. Red lines indicate the prodigiosin production and blue lines indicate biomass (OD_600_). JNB5-1 is a prodigiosin producing strain isolated form soil samples. PG-1 is a prodigiosin producing recombinant strain constructed by overexpression of *psrA* gene under the control of its own promoter P*_PsrA_ via* the plasmid pUCP18 in strain JNB5-1. PG-2 is a prodigiosin producing recombinant strain constructed by overexpression of *ompR* gene under the control of its own promoter P*_OmpR_ via* the plasmid pUCP18 in strain JNB5-1. PG-3 is a prodigiosin producing recombinant strain constructed by overexpression of *ompR* gene under the control of its own promoter P*_OmpR_* and *psrA* gene under the control of its own promoter P*_PsrA_ via* the plasmid pUCP18 in strain JNB5-1. PG-4 is a prodigiosin producing recombinant strain constructed by overexpression of *psrA* gene under the control of the promoter P17 (P*_RplJ_*) *via* the plasmid pUCP18 in strain JNB5-1. PG-5 is a prodigiosin producing recombinant strain constructed by overexpression of *ompR* gene under the control of the promoter P17 (P*_RplJ_*) *via* the plasmid pUCP18 in strain JNB5-1. PG-6 is a prodigiosin producing recombinant strain constructed by overexpression both of *ompR* and *psrA* genes under the control of the promoter P17 (P*_RplJ_*) *via* the plasmid pUCP18 in strain JNB5-1. PsrA and OmpR are two prodigiosin synthesis activators in strain JNB5-1. The experiments were performed in biological triplicates. Error bars indicate the standard deviations. One-way analysis of variance (ANOVA) was used to examine the mean differences between the data groups. ****p < 0.001; ns, no significance difference.

In recent years, transcription factor engineering has gained much attention due to its efficient application in altering gene transcription and improve the yield of target product (Li et al., 2018, 2021; Deng et al., 2021). PsrA (Pan et al., 2022) and OmpR are two prodigiosin synthesis activators in strain JNB5-1 we have identified by Tn5G transposon insertion mutation. To efficiently production of prodigiosin, *psrA* and *ompR* genes, driven by its own promoter, were cloned into pUCP18 plasmid, and transformed into strain JNB5-1 to construct strain PG-1 and PG-2, respectively. Shake flask fermentation in fermentation medium showed that the yield of prodigiosin produced by recombinant strains PG-1 and PG-2 was 8.36 g/L and 7.92 g/L, respectively. These yields were 132.07% and 125.12% that of wild-type strain JNB5-1 (6.33 g/L), respectively (Figure 4). Furthermore, a recombinant strain PG-3 expression both the *psrA* and *ompR* genes driven by their own promoter was constructed, and shake flask fermentation analysis showed that the PG-3 strain could production of 8.74 g/L of prodigiosin, which was 138.07% that of strain JNB5-1 (Figure 4). These results further confirmed that PsrA and OmpR are prodigiosin synthesis activators in strain JNB5-1 and transcription factor engineering could improve the yield of prodigiosin production.

As we can see, promoter engineering was widely used to enhance target metabolites production in other microorganisms (Deng et al., 2018; Ma et al., 2018; Liu et al., 2022). Hence, we then tried to further improve the prodigiosin production in strain JNB5-1 by promoter engineering. The strong promoter P17 identified in our study was selected to substitute the P*_PsrA_* and P*_OmpR_* promoters in strains PG-1, PG-2, and PG-3, and recombinant strains PG-4, PG-5 and PG-6 were obtained, respectively. Shake flask fermentation in fermentation medium showed that identical with strains JNB5-1, PG-1, PG-2, and PG-3, after 72 h of fermentation, the yield of prodigiosin produced by strains PG-4, PG-5, and PG-6 reached the highest value, of 9.36 g/L, 9.05 g/L, and 10.25 g/L, respectively. Among them, the strain PG-6 showed highest yield of prodigiosin, and was 161.93% that of wild-type strain JNB5-1 (6.33 g/L) and 117.28% that of recombinant strain PG-3 (8.74 g/L; Figure 4). This result suggested that using transcription factor engineering and promoter engineering can simultaneously improve the prodigiosin production in strain JNB5-1.

## Discussion

*Serratia marcescens*, a Gram-negative rod-shaped bacterium of the Enterobacteriaceae family, is found in a wide range of ecological niches and can produce many high-value secondary metabolites like prodigiosin (Williamson et al., 2006a), althiomycin (Gerc et al., 2014), serratamolide (Shanks et al., 2012), acetoin (Gao et al., 2014), and 2,3-butanediol (Rao et al., 2012). Among them, prodigiosin has received widespread attention due to its antimalarial, antibacterial, antifungal, antiprotozoal and immunosuppressant activities (Williamson et al., 2006a). Besides the well-studied *pigA* (encoding acyl-CoA dehydrogenase PigA), *pigB* (encoding FAD-dependent oxidoreductase PigB), *pigC* (encoding PEP-utilizing enzyme PigC), *pigD* (encoding prodigiosin biosynthesis protein PigD), *pigE* (encoding aminotransferase PigE), *pigF* (encoding O-methyl transferase PigF), *pigG* (encoding peptidyl carrier protein PigG), *pigH* (encoding aminotransferase PigH), *pigI* (encoding L-prolyl-AMP ligase PigI), *pigJ* (encoding beta-ketomyristol-ACP synthase PigJ), *pigK* (encoding prodigiosin biosynthesis protein PigK), *pigL* (encoding 4′-phosphopantetheinyl transferase PigL), *pigM* (encoding prodigiosin biosynthesis protein PigM), and *pigN* (encoding oxidoreductase PigN) genes involved in the metabolic pathway of the prodigiosin (Williamson et al., 2006a), lots of transcriptional regulator-encoding genes that play important roles in prodigiosin synthesis in *S. marcescens* have also been investigated, such as negative regulators MetR (Pan et al., 2020), SpnR (Horng et al., 2002), CopA (Williamson et al., 2006b), CRP (Stella and Shanks, 2014), RssB (Horng et al., 2010), RcsB (Brothers et al., 2019; Pan et al., 2021), CpxR (Sun et al., 2020) and SmaR (Coulthurst et al., 2006), and positive regulators EepR (Shanks et al., 2017), PigP (Shanks et al., 2013), GumB (Stella et al., 2018), RbsR (Lee et al., 2017), RpoS (Qin et al., 2020), and PsrA (Pan et al., 2022). However, our understanding of the regulatory mechanisms behind prodigiosin synthesis in *S. marcescens* is still limited. In this study, through transposon insertion mutation and genetic experiment (Figure 1), two-component system response regulator OmpR was confirmed to function as a prodigiosin activator in *S. marcescens* JNB5-1. This consistent with the result that shake flask fermentation analysis showed that the yield of prodigiosin produced by *ompR* disrupted mutant SK6-35 (10.21 mg/L) was only 0.20 times that synthesized by wild-type strain JNB5-1 (51.22 mg/L) in LB medium and the yield of prodigiosin produced by *ompR* overexpressed strain PG-2 (7.92 g/L) was 125.12% that synthesized by wild-type strain JNB5-1 (6.33 g/L) in fermentation medium (Figures 1B, 4).

Also, due to its important antimalarial, antibacterial, antifungal, antiprotozoal and immunosuppressant activities, lots of studies have been done to improving prodigiosin production through optimization of fermentation parameters of *S. marcescens* in the past, such as media composition (Chang et al., 2011) and pH (Fender et al., 2012), temperature (Elkenawy et al., 2017), and incubation period (Elkenawy et al., 2017). However, it is still a challenge for high-efficiency production of prodigiosin for commercial purposes. Promoter engineering and transcription factor engineering are two methods widely used to improve the yield of the target product in recent years, such as 3-aminopropionic acid (Song et al., 2015) and myo-inositol (Tang et al., 2020) in *E. coli*, Menaquinone-7 in *B. subtilis*, L-lysine (Huang et al., 2021), N-acetylglucosamine (Deng et al., 2021) and L-proline (Liu et al., 2022) in *C. glutamicum*. In this study, to increase the production of prodigiosin, endogenous promoters from *S. marcescens* were screened using RNA-Seq analysis, reporter green fluorescent protein analysis and RT-qPCR analysis (Figures 2, 3). Results showed the promoter P17 (P*_RplJ_*) of *rplJ* gene was found to be a strong constitutive promoter in strain JNB5-1. Finally, promoter engineering and transcription factor engineering was used to improving prodigiosin production in *S. marcescens* JNB5-1, and a strain PG-6 was obtained. Results showed the prodigiosin titer of this strain was increased to 10.25 g/l, which was 1.62-times that of the original strain JNB5-1 (6.33 g/l; Figure 4). As far as we know, our study is the first well-characterized constitutive promoter library from *S. marcescens*, and the transcription factor engineering and promoter engineering can be also useful strategies to improve the production of other high value-added products in *S. marcescens*.

In summary, this work describes a novel regulator OmpR which controls prodigiosin synthesis in *S. marcescens* JNB5-1 and shows a method improving prodigiosin production in *S. marcescens* by transcription factor engineering and promoter engineering. Further research is needed to reveal the molecular mechanisms how OmpR regulate prodigiosin production in *S. marcescens* JNB5-1.

## Data availability statement

The original contributions presented in the study are included in the article/Supplementary material, further inquiries can be directed to the corresponding author.

## Author contributions

XP performed the experiments and wrote the manuscript. JY and MT helped to wrote the manuscript. XZ, MX, and TY contributed to the analysis design and data interpretation. ZR designed the experiments and wrote the manuscript. All authors contributed to the article and approved the submitted version.

## Funding

This work was supported by the National Natural Science Foundation of China (32100055 and 31870066), the National Key Research and Development Program of China (2021YFC2100900), the Natural Science Foundation of Jiangsu Province (BK20210464), the project funded by China Postdoctoral Science Foundation (2021M691280), the Jiangsu Planned Projects for Postdoctoral Research Funds (2021K296B), and the Fundamental Research Funds for the Central Universities (JUSRP12119).

## Conflict of interest

The authors declare that the research was conducted in the absence of any commercial or financial relationships that could be construed as a potential conflict of interest.

## Publisher’s note

All claims expressed in this article are solely those of the authors and do not necessarily represent those of their affiliated organizations, or those of the publisher, the editors and the reviewers. Any product that may be evaluated in this article, or claim that may be made by its manufacturer, is not guaranteed or endorsed by the publisher.
