# Supplementary material for: Improving prodigiosin production by transcription factor engineering and promoter engineering in Serratia marcescens
Source: Front Microbiol. 2022 Aug 3;13:977337. doi: 10.3389/fmicb.2022.977337 (PMC9382025; doi:10.3389/fmicb.2022.977337)
Supplement: Supplementary file 1 [file Data_Sheet_1.PDF]

Improving Prodigiosin Production by Transcription factor Engineering and Promoter Engineering in *Serratia marcescens*

Xuewei Pan[1], Jiajia You[1], Mi Tang[1], Xian Zhang[1], Meijuan Xu[1], Taowei Yang[1], Zhiming Rao[1]*

1. Key Laboratory of Industrial Biotechnology of the Ministry of Education, Laboratory of Applied Microorganisms and Metabolic Engineering, School of Biotechnology, Jiangnan University, Wuxi 214122, China.

* Corresponding author: Zhiming Rao, E-mail: raozhm@jiangnan.edu.cn, Tel: 86-510-85916881.

Fig. S1

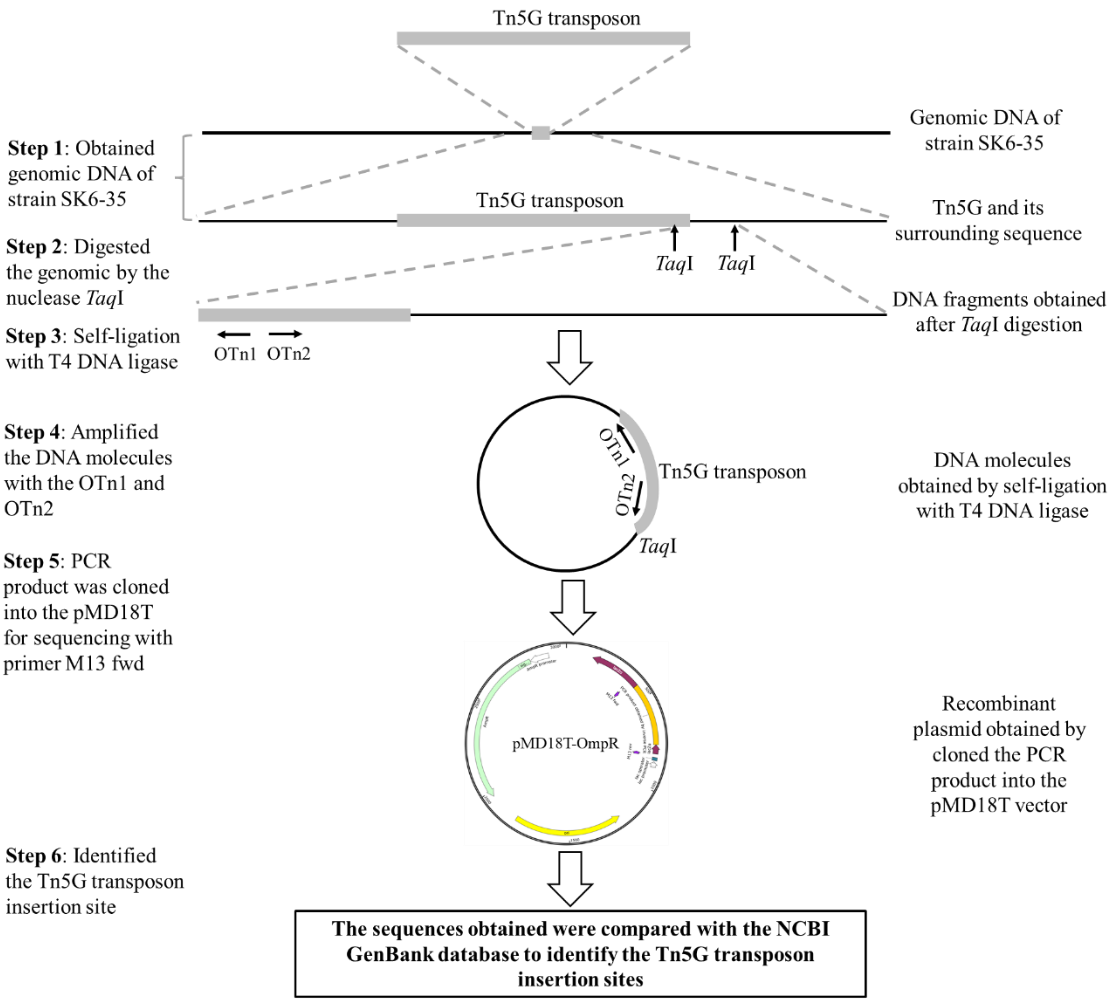

Figure S1. Schematic diagram of Tn5G insertion site in mutant SK6-35 analyzed by inverse PCR.

Table S1. Primers used in this study

Primer	Primer Sequence (5'-3')	Function
OTn1	GATCCTGGAAAACGGGAAAG	Identification of Tn5G in prodigiosin production
OTn2	CCATCTCATCAGAGGGTAGT	mutants
OmpR-F1	AAAACGACGGCCAGTGCCAAGCTTTCATGCCTTGCTGCCGTCC	Amplification of *ompR* gene to constructing of
OmpR-R1	TTCGAGCTCGGTACCCGGGGATCCATGCAAGAGAATCATAAGATCCTGGTTGTC	plasmid pXW2010
OmpR-arm-F1	TAGGCCGAATTCGAGCTCGGTACCGATGCACGTTCATCATCAGTTCGC	Amplification of *ompR* gene upstream
OmpR-arm-R1	AAACAAATAGGGGTTCCGCGATGAGGCGATTGCGCTTTTCAC	homologous arms
OmpR-arm-F2	GAAAAGCGCAATCGCCTCATCGCGGAACCCCTATTTGTTTATTTTTCT	Amplification of *aacC3* gene
OmpR-arm-R2	AAGCCTTTGGGAGTAATACCTCAGCCAATCGACTGGCGAG	
OmpR-arm-F3	CTCGCCAGTCGATTGGCTGAGGTATTACTCCCAAAGGCTTTATTGCCT	Amplification of *ompR* gene downstream
OmpR-arm-R3	ATTACAGCCGGATCCCCGGGTACCAACGTGAAAATCCCGGCCG	homologous arms
18-egfp-F1	AAAACGACGGCCAGTGCCAAGCTTCTATTTGTATAGTTCATCCATGCCATGTGTAATCC	Primers used to construct of plasmid pUCP18-
18-egfp-R1	TTCGAGCTCGGTACCCGGGGATCCATGAGTAAAGGAGAAGAACTTTTCACTGGAG	*egfp*
18-egfp-F2	GCCTGGGGTGCCTAATGAGT	Primers used to amplify the skeleton of plasmid
18-egfp-R2	ATGAGTAAAGGAGAAGAACTTTTCACTGGAGT	pUCP18-*egfp*
18-ahpC-F1	TTCTTCTCCTTTACTCATAATTACTCCTGTAATAGGTGTTAAAGGGTGGATG	Primers used to amplify the P1 (P_{AhpC}) promoter

18-ahpC-R1	TCATTAGGCACCCCAGGCGGTCAAGCCCAAGCGTTTGAAAT	
18-clpP-F1	AGTTCTTCTCCTTTACTCATTACCGTCTCCTAATAGAAATTGCCTTGGC	Primers used to amplify the P2 (P_{ClpP}) promoter
18-clpP-R1	ACTCATTAGGCACCCCAGGCGAACCGGCGCACGATGAAAAAG	
18-cpxP-F1	AGTTCTTCTCCTTTACTCATTTCAACTCCTCAAGCTTTCTCACTTTGCG	Primers used to amplify the P3 (P_{CpxP}) promoter
18-cpxP-R1	ACTCATTAGGCACCCCAGGCTATGATTTACCTCCAGACGCAAAATACGTCATC	
18-cspC-F1	AGTTCTTCTCCTTTACTCATTTGTGTTTCCTTTACTTGATAAACCTGCCTTG	Primers used to amplify the P4 (P_{CspC}) promoter
18-cspC-R1	ACTCATTAGGCACCCCAGGCAGTTTCGTACCTGGATCGGTGAAAG	
18-eno-F1	AGTTCTTCTCCTTTACTCATTAGGTTTTCCTCAGTACAAGTTAAACTAAAACTCCAG	Primers used to amplify the P5 (P_{Eno}) promoter
18-eno-R1	ACTCATTAGGCACCCCAGGCCATATTTGGTAGGCGGCGCG	
18-ftsZ-F1	AGTTCTTCTCCTTTACTCATAGTTTCTCTCCGTTTTGTGCCTGTC	Primers used to amplify the P6 (P_{FtsZ}) promoter
18-ftsZ-R1	ACTCATTAGGCACCCCAGGCTGTTTCAACAAAGAGATCATGCTGAGTAATCTT	
18-gltA-F1	AGTTCTTCTCCTTTACTCATTTACTGTCTCCTTAGCGCCTTATTTTAAAAACC	Primers used to amplify the P7 (P_{GltA}) promoter
18-gltA-R1	ACTCATTAGGCACCCCAGGCACAGCTCTTATTATTTTCCTTCCTCCGG	
18-lpp-F1	AGTTCTTCTCCTTTACTCATTATAACACCCTCTAGATTGAGTTAATCTCCATGTAGC	Primers used to amplify the P8 (P_{Lpp}) promoter
18-lpp-R1	ACTCATTAGGCACCCCAGGCTATAAAACGTGACATAATTGTCTTGATCAAAACGCC	
18-lpxC-F1	AGTTCTTCTCCTTTACTCATCGTTTTATCTCGCAATGTTATCCATCCTACC	Primers used to amplify the P9 (P_{LpxC}) promoter
18-lpxC-R1	ACTCATTAGGCACCCCAGGCGAATATCCTGAGAATTTGGAATCTCCGCTCT	

18-nlpI-F1	AGTTCTTCTCCTTTACTCATTTCCCACTCCCGAAGACAAACAT	Primers used to amplify the P10 (P_{NlpI}) promoter
18-nlpI-R1	ACTCATTAGGCACCCCAGGCCTGTATAGATAGTTTTACAGCTCCCCGC	
18-ompA-F1	AGTTCTTCTCCTTTACTCATTTTGCGCCTCGTTATCATCCAAAT	Primers used to amplify the P11 (P_{OmpA}) promoter
18-ompA-R1	ACTCATTAGGCACCCCAGGCGTAAATTTAGGATTTTTCCCATCATTTTCGCACC	
18-ompC-F1	AGTTCTTCTCCTTTACTCATCGTTATTATCCTCGTTAATTATGTCGAGCTACG	Primers used to amplify the P12 (P_{OmpC}) promoter
18-ompC-R1	ACTCATTAGGCACCCCAGGCGAATTTTACTCGCTGAAGGTGCGC	
18-ompN-F1	AGTTCTTCTCCTTTACTCATCATTATTACCCTCATTGGTGTTATTCGGACAC	Primers used to amplify the P13 (P_{OmpN}) promoter
18-ompN-R1	ACTCATTAGGCACCCCAGGCTTTATACGATCACACGTTTTTTAAACGATTTCGTTACAAA	
18-ompW-F1	AGTTCTTCTCCTTTACTCATTATCCATTCCATTTGTGTGGTTAATCGCT	Primers used to amplify the P14 (P_{OmpW}) promoter
18-ompW-R1	ACTCATTAGGCACCCCAGGCCTTGATTGCCCACCGACTCCT	
18-ompX-F1	AGTTCTTCTCCTTTACTCATAACCACCTCAAACGCGTTTTATTATTAAGTACA	Primers used to amplify the P15 (P_{OmpX}) promoter
18-ompX-R1	ACTCATTAGGCACCCCAGGCCCTTAACGGGCCTTTTTTGCGC	
18-raiA-F1	AGTTCTTCTCCTTTACTCATATAACTTACCTCTCTGTCTTTCCGTCTTGG	Primers used to amplify the P16 (P_{RaiA}) promoter
18-raiA-R1	ACTCATTAGGCACCCCAGGCTCGGCAACGGCAGAAAAACCA	
18-rplJ-F1	AGTTCTTCTCCTTTACTCATTAGCTTTTTGCTCCTGGATTAGCCG	Primers used to amplify the P17 (P_{RplJ}) promoter
18-rplJ-R1	ACTCATTAGGCACCCCAGGCTCGCACTTGCGATTATCGCTTTG	
18-rpoB-F1	AGTTCTTCTCCTTTACTCATAGGGTTCCTCAGCTCGCTGA	Primers used to amplify the P18 (P_{RpoB}) promoter

18-rpoB-R1	ACTCATTAGGCACCCCAGGCGCCAACCTTTCCGGTTGCAG	
18-rpoH-F1	AGTTCTTCTCCTTTACTCATTCAAACCCTCTCTATGAGAATACAAAATCATGCAG	Primers used to amplify the P19 (P_{RpoH}) promoter
18-rpoH-R1	ACTCATTAGGCACCCCAGGCGATTTTTTTGGTATACTCTTCTCCTGCTGCT	
18-rpsA-F1	AGTTCTTCTCCTTTACTCATGTTGTTAATCTTCAGGGTTCTTTAGTTTAACGTCCA	Primers used to amplify the P20 (P_{RpsA}) promoter
18-rpsA-R1	ACTCATTAGGCACCCCAGGCGCGGCGCTATTGGCTTTTTGTC	
18-rpsF-F1	AGTTCTTCTCCTTTACTCATCGAATTGCTCCTTACGGATTATTCAGCC	Primers used to amplify the P21 (P_{RpsF}) promoter
18-rpsF-R1	ACTCATTAGGCACCCCAGGCAGTGCCTCTCTTACTTTTTGCCGT	
18-rpsM-F1	AGTTCTTCTCCTTTACTCATTATGCACTCCTACTATTTTATACAGCAACACCATTCT	Primers used to amplify the P22 (P_{RpsM}) promoter
18-rpsM-R1	ACTCATTAGGCACCCCAGGCTTATCTCGCATATTTTTCTTGCAAAGTTGGGTTGA	
18-12140-F1	AGTTCTTCTCCTTTACTCATGCGAACCTCCTTAAAAACGCCTG	Primers used to amplify the P23 ($P_{SMWW4\_v1c12140}$)
18-12140-R1	ACTCATTAGGCACCCCAGGCGGGACACCTCCAGAGGTGTTG	promoter
18-29250-F1	AGTTCTTCTCCTTTACTCATAGTGGCACCTTACAGTTTGTTTCACC	Primers used to amplify the P24 ($P_{SMWW4\_v1c29250}$)
18-29250-R1	ACTCATTAGGCACCCCAGGCAGCCGAAATGACGAGGCG	promoter
18-sodB-F1	AGTTCTTCTCCTTTACTCATTGCTTCCTCCTTTACAGCGCC	Primers used to amplify the P25 (P_{SodB}) promoter
18-sodB-R1	ACTCATTAGGCACCCCAGGCTTCCCTGTTCTGCGCGGT	
18-tpiA-F1	AGTTCTTCTCCTTTACTCATGTTTTTTCCTCCAACTAGGGAACGC	Primers used to amplify the P26 (P_{TpiA}) promoter
18-tpiA-R1	ACTCATTAGGCACCCCAGGCCGGCGCGGCATTTAATGAC	

18-trxA-F1	AGTTCTTCTCCTTTACTCATGTTCTACTCCACAGGATTATGTCTACCTTGT	Primers used to amplify the P27 (P_{TrxA}) promoter
18-trxA-R1	ACTCATTAGGCACCCCAGGCAGTGTGGTAGAATATCAGCTAACTATTGCTTTACG	
18-uspG-F1	AGTTCTTCTCCTTTACTCATATCACGTCCTTTCTATGTAAACCGGG	Primers used to amplify the P28 (P_{UspG}) promoter
18-uspG-R1	ACTCATTAGGCACCCCAGGCTCTCGAGTAATAAATAATTAATCGCCTATCCGTTATCG	
18-yabY-F1	AGTTCTTCTCCTTTACTCATGGATCGGTCTCCTTTTTTATTATCAACATGTTGG	Primers used to amplify the P29 (P_{YabY}) promoter
18-yabY-R1	ACTCATTAGGCACCCCAGGCAAAGTTGTTACCTTTCTAATAATCATTTTGCGCAGT	
18-yccA-F1	AGTTCTTCTCCTTTACTCATAATGCTCTCTCTTATCAGGCCATCACAAAATAATC	Primers used to amplify the P30 (P_{YccA}) promoter
18-yccA-R1	ACTCATTAGGCACCCCAGGCGCAACGACGCGCACTATAAACAG	
18-yfiD-F1	AGTTCTTCTCCTTTACTCATTATGTTGCCTCCGTACAAGGGC	Primers used to amplify the P31 (P_{YfiD}) promoter
18-yfiD-R1	ACTCATTAGGCACCCCAGGCAAACGCTCTCCTTTGTTTTCTTTAGCAATTC	
18-ygdI-F1	AGTTCTTCTCCTTTACTCATTGAGGACTCCTTTGTAGTTTTAAATGCTGATAAATACC	Primers used to amplify the P32 (P_{YgdI}) promoter
18-ygdI-R1	ACTCATTAGGCACCCCAGGCACCCTGATTGTCACTGGCTTGAG	
18-AmpR-F1	AGTTCTTCTCCTTTACTCATACTCTTCCTTTTTCAATATTATTGAAGCATTTATCAGGG	Primers used to amplify the AmpR promoter
18-AmpR-R1	ACTCATTAGGCACCCCAGGCCGCGGAACCCCTATTTGTTTATTTTTCTAAATAC	
eGFP-F1	TGCCATGCCCGAAGGTTA	qPCR primers, coding region of gene *egfp*
eGFP-R1	CGTGTCTTGTAGTTCCCGTCATC	
16S rRNA-F1	CACACCGCCCGTCACACCA	qPCR primers, coding region of gene 16S rRNA

16S rRNA-R1	CGCAGGTTCCCCTACGGTTAC	
18-PompR-ompR-F1	AAAACGACGGCCAGTGCCAAGCTTTCATGCCTTGCTGCCGTCCG	Primers used to amplify the *ompR* gene under the
18-PompR-ompR-R1	TTCGAGCTCGGTACCCGGGGATCCCCCGACGGCTAAACGCCACC	control of its own promoter
18-PpsrA-PsrA-F1	GTAAAACGACGGCCAGTGCCAAGCTTTCAGGCAGAGTGATACTGTTCAGTAATCAG	Primers used to amplify the *psrA* gene under the
18-PpsrA-PsrA-R1	AATTCGAGCTCGGTACCCGGGGATCCGGGCCTTGGTGGTTCAACCAC	control of its own promoter
18-PompRpsrA-ompRpsrA-F1	GTAAAACGACGGCCAGTGCCAAGCTTTCATGCCTTGCTGCCGTCCG	Primers used to amplify the *psrA* and *ompR* genes
18-PompRpsrA-ompRpsrA-R1	AACAGTATCACTCTGCCTGACCCGACGGCTAAACGCCACC	under the control of their own promoters
18-PompRpsrA-ompRpsrA-F2	GGTGGCGTTTAGCCGTCGGGTCAGGCAGAGTGATACTGTTCAGTAATCAG	
18-PompRpsrA-ompRpsrA-R2	AATTCGAGCTCGGTACCCGGGGATCCGGGCCTTGGTGGTTCAACC	
18-P17-ompR-F1	GTAAAACGACGGCCAGTGCCAAGCTTTCATGCCTTGCTGCCGTCCG	Primers used to amplify the *ompR* gene under the
18-P17-ompR-R1	AATCCAGGAGCAAAAAGCTAATGCAAGAGAATCATAAGATCCTGGTTGTCGATG	control of the P17 promoter
18-P17-ompR-F2	ATCTTATGATTCTCTTGCATTAGCTTTTTGCTCCTGGATTAGCCGG	
18-P17-ompR-R2	AATTCGAGCTCGGTACCCGGGGATCCTCGCACTTGCGATTATCGCTTTG	
18-P17-psrA-F1	GTAAAACGACGGCCAGTGCCAAGCTTTCAGGCAGAGTGATACTGTTCAGTAATCAG	Primers used to amplify the *psrA* gene under the
18-P17-psrA-R1	AATCCAGGAGCAAAAAGCTAATGCCCGTAAATTTTGATCTCAACGATCT	control of the P17 promoter
18-P17-psrA-F2	AGATCAAAATTTACGGGCATTAGCTTTTTGCTCCTGGATTAGCCG	
18-P17-psrA-R2	AATTCGAGCTCGGTACCCGGGGATCCTCGCACTTGCGATTATCGCTTTG	

18-P17-ompRpsrA-F1	GTAAAACGACGGCCAGTGCCAAGCTTTCAGGCAGAGTGATACTGTTCAGTAATCAG	Primers used to amplify the *ompR* and *psrA* gene
18-P17-ompRpsrA-R1	CGGACGGCAGCAAGGCATGAATGCCCGTAAATTTTGATCTCAACGATCT	under the control of the P17 promoter
18-P17-ompRpsrA-F2	AGATCAAAATTTACGGGCATTCATGCCTTGCTGCCGTCCG	
18-P17-ompRpsrA-R2	AATCCAGGAGCAAAAAGCTAATGCAAGAGAATCATAAGATCCTGGTTGTCGATGA	
18-P17-ompRpsrA-F3	ATCTTATGATTCTCTTGCATTAGCTTTTTGCTCCTGGATTAGCCG	
18-P17-ompRpsrA-R3	AATTCGAGCTCGGTACCCGGGGATCCTCGCACTTGCGATTATCGCTTTG	

Table S2. The 61 genes that highly expressed under all four conditions

Gene	Average FPKM	Product
ompA	21640.7025	Outer membrane protein A
hupA	9812.94	DNA-binding transcriptional regulator, alpha subunit
ompW	5126.42	Outer membrane protein W
ompN	10778.6525	Outer membrane pore protein
ybaY	8141.1775	Outer membrane lipoprotein
raiA	9965.2425	Cold shock protein associated with 30S ribosomal subunit
ompX	6679.55	Outer membrane protein X
ompC	4732.635	Outer membrane porin protein C
gapA	5062.2025	Glyceraldehyde-3-phosphate dehydrogenase A
cspC	8650.905	Stress protein, member of the CspA-family
SMWW4_v1c29250	2818.635	Hypothetical protein
ahpC	3612.45	Alkyl hydroperoxide reductase, C22 subunit
sodB	2468.105	Superoxide dismutase
lpp	8164.8725	Murein lipoprotein
gltA	2364.4775	Citrate synthase
pgk	2444.4925	Phosphoglycerate kinase
rplJ	5684.93	50S ribosomal subunit protein L10
rpsF	5404.0175	30S ribosomal subunit protein S6
rseA	2895.525	Sigma-E factor negative regulatory protein RseA
uspG	1753.1375	Universal stress protein UP12
yfiD	3249.205	Autonomous glycyl radical cofactor
uspA	2927.7325	Universal stress global response regulator
SMWW4_v1c12140	1975.2125	Hypothetical protein
lpxC	2541.38	UDP-3-O-acyl N-acetylglucosamine deacetylase
rpsD	4388.445	30S ribosomal subunit protein S4
ftsZ	1703.47	Cell division protein FtsZ

rpsM	5304.5475	30S ribosomal subunit protein S13
tpiA	2013.625	Triosephosphate isomerase
SMWW4_v1c16980	4148.5875	CspA family cold shock transcriptional regulator
rpoS	2251.38	RNA polymerase, sigma S (sigma 38) factor
rplF	3818.43	50S ribosomal subunit protein L6
rpsE	4551.18	30S ribosomal subunit protein S5
rpoA	2810.17	RNA polymerase, alpha subunit
rpsR	3253.605	30S ribosomal subunit protein S18
rpsA	2618.3375	30S ribosomal subunit protein S1
ygdI	2606.52	Putative lipoprotein
eno	1959.8025	Enolase
rplE	3278.4675	50S ribosomal subunit protein L5
fusA2	2736.9575	Elongation factor G
hupB	3855.9675	DNA-binding protein HU-beta
rplM	2622.89	50S ribosomal subunit protein L13
rplO	2843.77	50S ribosomal subunit protein L15
secY	2546.31	Preprotein translocase membrane subunit
ihfB	1780	Integration host factor (IHF)
rplC	2428.4275	50S ribosomal subunit protein L3
rpoH	1774.61	RNA polymerase, sigma 32 (sigma H) factor
pal	1621.7925	peptidoglycan-associated outer membrane lipoprotein
rpoB	1454.715	RNA polymerase, beta subunit
clpA	1163.19	ATP-dependent serine protease
rpsH	2558.6	30S ribosomal subunit protein S8
clpP	1144.5125	ATP-dependent Clp protease
skp	1289.2625	periplasmic chaperone
rpsJ	2430.1975	30S ribosomal subunit protein S10
trxA	1508.045	thioredoxin 1
rpsG	1823.7575	30S ribosomal subunit protein S7

hfq	1926.645	Host factor for RNA phage Q beta replication
arcA	1205.485	DNA-binding response regulator
yccA	1318.955	HflBKC-binding inner membrane protein
cspE	4084.85	DNA-binding transcriptional repressor
nlpI	1242.08	Lipoprotein
cpxP	1919.7225	Cpx response inhibitor
